# Insights on dental care management and prevention in children with autism spectrum disorder (ASD). What is new?

**DOI:** 10.3389/froh.2022.998831

**Published:** 2022-09-27

**Authors:** Nicoletta Zerman, Francesca Zotti, Salvatore Chirumbolo, Alessandro Zangani, Giovanni Mauro, Leonardo Zoccante

**Affiliations:** ^1^ Department of Surgery, Dentistry, Paediatrics and Gynecology, University of Verona, Verona, Italy; ^2^ Department of Neurosciences, Biomedicine and Movement Sciences, University of Verona, Verona, Italy; ^3^ Dentistry Private Practice, Mantova, Italy; ^4^ Autism Veneto Region Center, Azienda Ospedaliera Universitaria Integrata of Verona, Verona, Italy

**Keywords:** dentistry, autism spectrum disorder (ASD), prevention, oral health / hygiene, telehealth, music therapy and autism spectrum disorder, dentist education

## Abstract

Autistic subjects represent a severe concern to dentistry due to the considerable difficulty in managing their oral health, maintaining routine toothbrushing, and preventing dental and periodontal problems. The social and economic burden of managing dental care in autism spectrum disorder (ASD) children is particularly cumbersome for families and public and private health expenditure, especially when children reach the dentist following a late diagnosis with evident oral health problems. An early diagnosis of ASD helps dentists better address these children's oral health. Unfortunately, insufficient attention is paid to the training and education of general pediatricians, dentists, and dental hygienists, allowing them to get to approach the different clinical aspects of ASD. Usually, children diagnosed with ASD are scheduled for dental appointments like their neurotypical peers, whereas their needs are typically complex and personalized. Scant attention is also devoted to these patients by commercial manufacturers of dental products and devices for oral hygiene and prevention of caries and periodontal diseases, leaving parents without the support and often failing when they address the oral health of autistic children. The difficulties of oral care do not derive simply from the behavior of ASD patients, as is commonly assumed, and therefore cannot be overcome solely by the patience and attention of parents and dentists. Genetics, dietary habits, sensory impairments, and cognition disorders are other causes contributing in various degrees to the impact on the mood and psychological reactions of autistic children towards dentists. How can we prevent teeth caries, periodontal disorders, and other oral health impairments by properly managing ASD children? This manuscript gives an up-to-date overview of these problems and helps to provide good remarks.

## Introduction

Autism spectrum disorder (ASD) is a neurodevelopmental disorder mainly involving impairments in language, communication, and social interaction. The patient with ASD shows narrow interests, stereotyped and repetitive behaviors, including a set of different neurodevelopmental alterations linked to abnormal brain development that begins already in the fetal period, long before the child's birth (1–4).

Current literature reports that children affected by ASD usually experience unusual tooth decay and even teeth loss, when compared to neurotypical peers, and are more frequently prone to develop other dental health issues, including dental and soft-tissue traumas and teeth grinding (5–7).

Most of the literature in the field to date deals with oral hygiene in the autistic subject, focusing mainly on caries and periodontal disease as related to poor hygiene, while there is almost nothing or very few issues about prevention. As we will say further on in this review, to be effective, any preventive measure in dentistry should be undertaken very early, in the neonatal stage or even before, through complete information and educational assistance to pregnant women.

The recent meta-analysis performed by Nunes da Silva et al. on about 928 articles dating up to 2015 dealing with ASD in both adults and pediatric subjects reported that the pooled prevalence of dental caries in autistic children was 60.6% (CI_95_ = 44.0–75.19) while that of periodontal diseases was 69.4% (CI_95_ = 47.6–85.0) (5). Furthermore, the systematic review by Corridore et al. assessed that the higher incidence of dental impairments in ASD children was due to periodontal disease (4).

The majority of systematic reviews and meta-analyses we selected have yet a certain risk of bias in their performance, except for those ones dealing with poor oral health in ASD respect to peers. This assesses that, so far, the major concern felt in dentistry for ASD is oral health and periodontal diseases caused by poor oral hygiene. However, many further items are to be considered. In this paper we will address this point at issue.

### Research strategy

**Figure 1:**
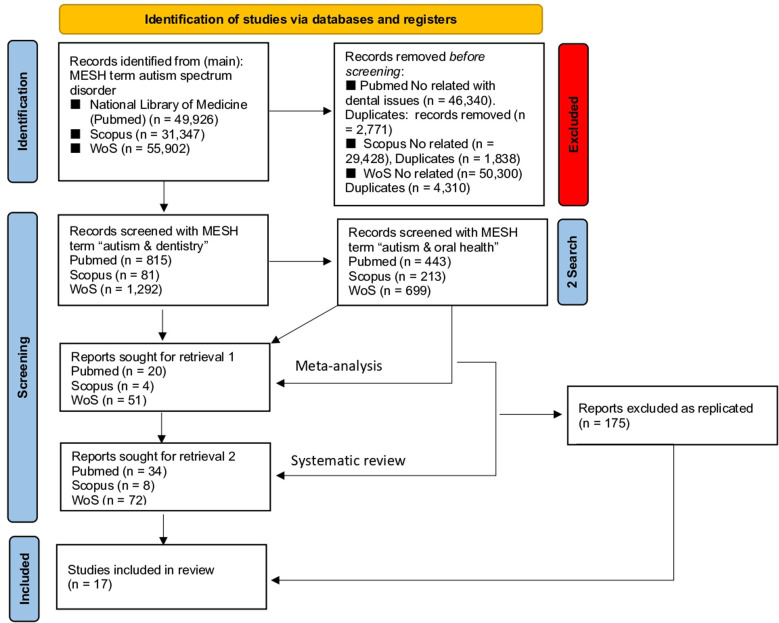
PRISMA model of our research study. See text for details.

Figure 1 describes the Preferred Reporting Items for Systematic Reviews and Meta-Analyses (PRISMA), which describes our research strategy on the current literature of the field.

**Figure 2:**
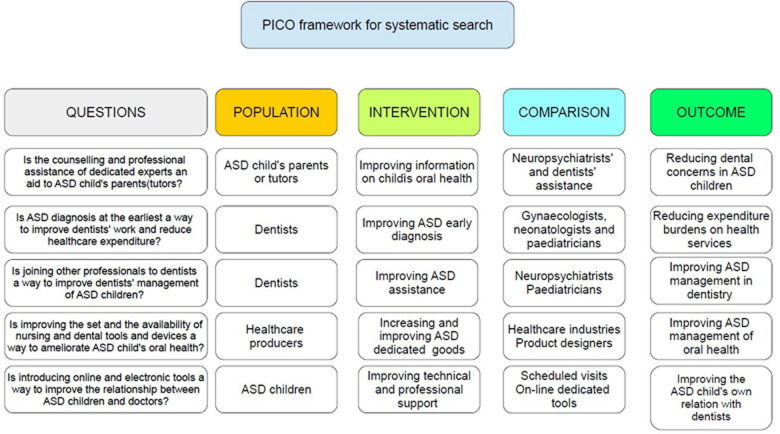
PICO framework of the search study.

The inclusion criteria for reporting the systematic review of systematic reviews includes all papers (both RCTs and NRSI) dealing directly or indirectly with the five fundamental questions we reported in the PICO framework (Figure 2), addressing: a) parents/tutors of ASD children; b) dentists; c) healthcare producers; d) ASD children and from which our search was planned and oriented (8). Searching was performed autonomously and independently by two of us (NZ and SC). Initially, the calculated Cohen's *K* resulted as % of agreement = 83.84% and Cohen's *k* = 0.38328. Disagreement (only the first wanted to include 20 items and only the second wanted to include 6 items) was resolved by a second and third tour of selection. So, Cohen's *K* finally resulted in: % of agreement = 88.07% and Cohen's *k* = 0.5018. A critical appraisal tool for systematic reviews that include randomized or non-randomized studies of healthcare interventions, or both (AMSTAR-2 checklist) has been added in this manuscript (9) (Table 2). SPSS v.26.0 was used for statistics.

A thorough search on the major literature databases on autism spectrum disorder, i.e. Pubmed/Medline, Scopus and Web of Science (WoS) was performed on June 30th 2022 and retrieved these results: National Library of Medicine (Pubmed) (*n* = 49,926), Scopus (*n* = 31,347), WoS (*n* = 55,902). Of these items, a number of issues were removed (see Figure 1).

**Figure 3:**
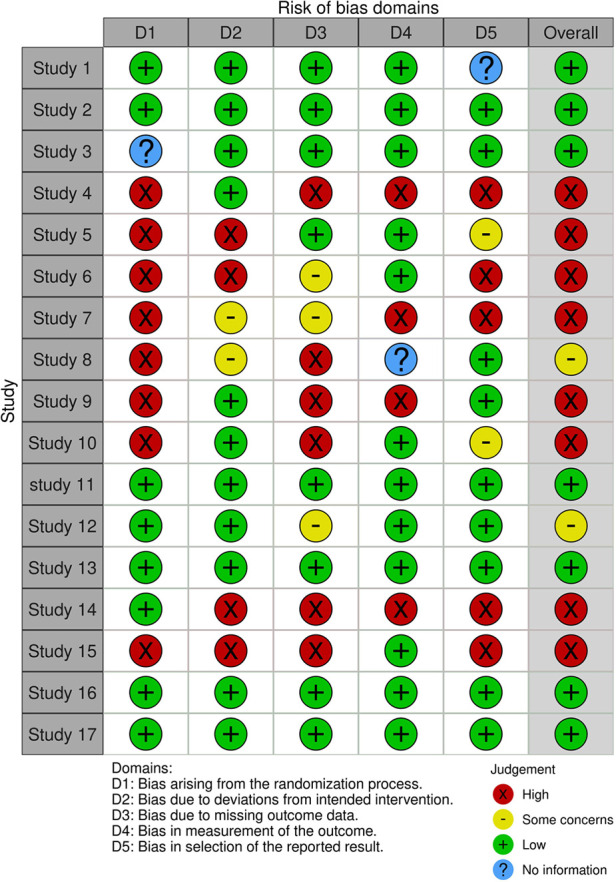
Rob (risk of bias) for the studies reported in Table 1.

Furthermore, a Risk of bias (using a ROB-2 tool) for each selected paper was performed (Figure 3) (10).

**Table 1:** Recent meta-analyses and systematic reviews on dentistry/oral health in autism.

Type of study	Total survey	Studies recruited	Study object	Results/reported evidence	References
Meta-analysis and systematic review	379 papers	37 papers	Visual pedagogy in ASD and dentistry	Visual pedagogy improves oral hygiene skills and cooperative actions during dental care	(11)
Meta-analysis	258 studies (391 cases)	8 studies	Oral health in ASD children	ASD is associated with poorer oral hygiene, an increase in the risk of caries, and a lower salivary pH than neurotypical peers	(12)
Meta-analysis	533 papers	9 studies (533 children)	Dental caries in ASD children	ASD associated with a worse dental health status than healthy neurotypical peers	(13)
Meta-analysis	1,437 records	15 studies	Oral health status in ASD children	The authors sustain that other factors besides ASD, such as sugar enriched diet and scant oral hygiene concur to worsening oral health	(14)
Systematic review	59 studies	9 studies retrieved	Studies on oral health behaviors in ASD children and adolescents	Difficulties related to autism-caused social impairments and sensory sensitivities.	(15)
Meta-analysis and systematic review	437 reports	13 observational, 7 quantitative	Malocclusion in ASD children	ASD children have a higher risk of overjet, not for all the malocclusion types, due to a high heterogeneity	(16)
Systematic review	204 reports	5 eligible and included	Psychological techniques in dental care	Inconclusive results	(17)
Systematic review	5,572 studies, retrieved 1,751 reports	133 full text analysis, 15 selected	Bruxism	Inconclusive results due to paucity	(18)
Systematic review	2,240 reports	77 studies	Tooth grinding	Inconclusive results due to variability and heterogeneity	(7)
Systematic review	815 reports	29 studies (43,916 participants)	Oral health	Were considered as risk factors consumption of sweet snacks, public schools attendance, low maternal education level, and socioeconomic status	(19)
Systematic review	742 records	5 papers	Gingival health status	Higher gingival and plaque index in ASD children	(20)
Systematic review	1,287 records	19 articles	Virtual reality and dental smartphone	These devices are not widely used in dentistry	(21)
Meta-analysis and systematic review	2,105 records	20 articles	Oral health	Worse in special needs children including ASD children	(22)
Meta-analysis and systematic review	567 records	25 issues	Oral health	Worse hygiene associated with disabilities	(23)
Systematic review	69 records	13 selected	Oral disease	Inconclusive results due to the high recurrence to general anesthesia	(4)
Meta-analysis and systematic review	928 records	7 included	Oral health status	High dental caries and periodontal diseases in ASD children	(5)
Systematic review	586 records	10 included	Oral health and sensory disorders	Oral, gingival and/or periodontal hygiene is worse in ASD children	(24)

### Research keywords

Using the MESH term “autism / dentistry” we retrieved 815 reports in Pubmed, 81 in Scopus and 1,292 in WoS. A further selection for the term “meta-analysis” allowed us to retrieve 20 reports in Pubmed, 4 in Scopus and 51 in WoS, whereas the term “systematic reviews” captured 34 reports in Pubmed, 8 in Scopus and 72 in WoS.

Using the MESH term “autism / oral health”, we retrieved 443 reports in Pubmed, 213 in Scopus and 699 in WoS. Again, adding the term “meta-analysis” we obtained 20 reports in Pubmed, 24 in Scopus and 37 in WoS, whereas with the term “systematic review” the system released 34 papers in Pubmed, 27 in Scopus and 51 in WoS. Two of our co-authors independently searched the databases (SC and NZ) and reviewed each of the retrieved articles. After removing replicated papers and reports out of our selection criteria (n = 175 reports excluded as replicated), i.e. meta-analyses and/or systematic reviews dealing with “autism-dentistry and oral health”, we were able to include an eligible number of 17 meta-analyses and systematic reviews, finally summarized in Table 1 (4, 5, 7, 11–24).

### First survey on the literature

These findings suggest that information about oral health in ASD is scarce, and there is poor communication between dentists and pediatricians when dealing with autistic patients.

In our opinion, dentists should address at least two different areas of prevention in pediatric autism. The first (primary prevention) deals with a very early phase in the tooth development and anticipating the correct formation and optimal health of dental and periodontal structures. The other (secondary prevention) involves reducing maximally and hampering the impact of damage to teeth that is mainly due to poor oral health (caries, gingival and dental disorders, periodontal tissue and dental injuries) (25) and improving the role of dentists' intervention, especially by reducing more complex therapies that require sedation, general anesthesia, and hospitalization.

From this perspective, autistic subjects suffering from previously described alteration of the connective tissues (26), should be considered as having a higher rate of orthodontic concerns regarding their neurotypical peers in any dental care education (27).

Nevertheless, some issues about the dental health status of autistic children are controversial because some reports showed contradictory results, probably due to the very complex matter of the debate (13, 28, 29). The use of an AMSTAR-2 check list should allow the more proper selection of eligible studies to perform a reliable systematic review or meta-analysis (9). Risk of bias and other confounders may even depend on flaws in the research plan or in the management of selected papers, i.e., bias, statistical confounders and incorrect stratification, eligibility criteria, and diagnosis of the enrolled patients and controls, despite the existence of some recent meta-analyses and systematic reviews allowed us to focus onto the debated problem (Table 1).

The relative paucity of reliable surveys, meta-analyses, and systematic reviews on autism in dentistry reported on Table 1, i.e., regarding oral hygiene and dentistry in autistic children, might be caused by the extreme difficulty in managing these subjects and because there is scant interest in addressing these crucial issues to date.

A first consideration to be held in mind is the correctness of the study rationale and statistics before starting any case/control investigation of the dental health of autistic subjects, and soundness can be achieved if the experimental setting is standardized. This stands as a very burdensome task if dealing with autistic patients. Therefore, in an observational case-control study, a correct, standardized clinical protocol to investigate the pathophysiology of autism dentistry is particularly burdensome, yet necessary.

The extreme difficulty that dentists exhibit in managing autistic children represents a further concern for performing a proper clinical investigation. Strange lights, noises, tastes, and smells, which are frequently found in a dentistry healthcare service, can be significant concerns to overcome for autistic patients.

In this context, it is crucial to be fully aware and adequately skilled in recognizing children's “hits,” i.e., triggering items causing the child's paroxysmal, nervous or uncontrolled behavior, usually managed by ASD subjects' caregivers. Furthermore, these signs of distress are usually present before the child's meltdown, usually referred to as a “rumble stage” (30, 31). This should also suggest that the primary goal of dentists, with the aid of other professionals, is to make children with these disorders feel comfortable. If the autistic child perceives that she/he is in a comfortable and fun environment, she/he is likely to feel safe when faced with health checks.

Moreover, in this perspective, the role of parents and dentists should be intertwined in a continuously encouraged cross-talk to assist ASD subjects in their oral hygiene correctly. Parents must be fully aware of their responsibility in dental health assistance to their autistic children (32, 33).

This evidence strongly recommends that parents or tutors not be left alone with this considerable concern, where parenting represents an emerging problem (34, 35). There is an urgent need to connect parents of ASD children with specific professionals, such as pediatric neuropsychiatrists, pediatricians, and caregivers before they access a dentistry service.

The autistic patient often shows the difficulty in assuming the recommendations and indications offered by dentistry professionals. Therefore, it is crucial to note that the most straightforward interplay between dentist and parents is insufficient to manage the autistic child correctly and that further highly skilled professionals and caregivers are fundamental in this sense.

It is well known that ASD children love their stereotyped and repetitive behaviors, so any unforeseen modification of routine habits, usually required when going to the dentist more than, for example, when going to the rehabilitation center or school, represents massive stress for them. A whole series of measures that can, in a certain sense, “calm and quiet the child,” even inducing “flourishing,” is therefore of the utmost importance for holding a proper clinical investigation (36).

One possibility might be the use of music as a therapy tool.

Recent evidence reported that music activates brain areas by increasing the oscillation synchrony between various cortical areas, thereby increasing sensory integration (37).

For this reason, music therapy has been suggested for ASD due to its intrinsic ability to modify both the structure and functional connectivity of the cortex (38). Furthermore, this improved multisensory integration among cortical areas seems to directly address the primary underlying neurophysiologic defect in the autistic patient (39). There are numerous studies on the topic (40–42). The Cochrane Collaboration 2014 revised its 2006 review on music and autism. Music therapy can help ASD children improve their skills in primary outcome areas that constitute the core of the condition, including social interaction, verbal communication, initiating behavior, and social-emotional reciprocity contributing in increasing social adaptation skills and promoting the quality of parent-child relationships.

ASD subjects show a bilateral activation of the temporal cortex while listening words in a song, in a similar way to the age and gender-matched control group (43).

For all these reasons, it seems reasonable to implement sung words (in a music listening) to the other cognitive-behavioral tools in the dental clinical management of ASD patients. Sometimes, the ASD subject cannot express her/his feelings and perceptions, such as fear or pain. Consequently, patient management becomes more and more complicated during the consultation unless using specific markers of children's feelings and behaviors (44–46). Oral care and hygiene in children are paramount, especially in autistic subjects more prone to caries, dental lesions, demineralization of the enamel, and bruxism (18, 47, 48).

These concerns can be prevented and avoided with periodic checks, perhaps focused on the increase in enamel strength, so it appears crucial to consult the dentist regularly (48–50). By the way, the many healthcare companies engaged in marketing dental products and devices for oral health are investing very little in more practical, comfortable, easy-to-use (and maybe eco-sustainable) toolkits for autistic subjects (51).

The clinical setting of an experimental trial to investigate the impact of ASD on dentistry and subjects' dental health is, therefore, particularly cumbersome from a methodological point of view. A first issue may be parents' perception of dental care in ASD children, which may provide suggestions for a better and more correct methodology in the clinical trials.

A recent survey by Duker et al. found four concerns among parents of ASD children, namely a) difficulty in finding the right dental expert able to operate with autistic children, b) the uncomfortable effect on children of sensory devices; c) the perception that any dental intervention appears as a kind of torture; d) mistrust about the drugs used in dentistry, a matter also examined in more recent articles (52–54). Therefore, prevention is always of utmost importance as it would mean the expenditure of money, time, and personal effort by parents, dentists, the social community, public healthcare systems, and providers.

How can we prevent tooth caries, periodontal disorders, and other oral health impairments in ASD children?

While dietary education is challenging to achieve, parents are much more motivated to look at regular dental appointments, trying to familiarize children with the dentist and her/his place of work. However, there is still insufficient consideration of these patients in health policy.

## Oral health prevention in the autistic subject as early as possible. Concerns and suggestions

When should preventive measures begin?

Early diagnosis of ASD is crucial for the correct prevention of oral health being undertaken at early as possible (55–57). Diagnosing autism during pregnancy is a possibility, particularly for women at risk (58, 59), yet dental assistance and counseling should be promoted to parents as soon as the possibility of autism spectrum disorder has been forecast (60, 61). As mentioned above, dentistry in ASD subjects is a significant concern for families and doctors. Hence an early autism diagnosis and pediatricians' preventive intervention, with the expert assistance of dentistry professionals and infancy neuropsychiatrists, in training the child for oral hygiene, should be part of the correct management of the autistic subject.

One issue is training dentists to distinguish the different spectra of ASD, i.e., whether classical autism or Asperger syndrome (299.00 DSM-IV), atypical autism (pervasive developmental disorder not otherwise specified, 299.80 DSM-IV), or Rett syndrome or more simply a non-autistic cognitive disorder (62). In addition, awareness of the existence of specific sensory modulation subtypes in autistic children, by sex and age, is crucial to address these patients appropriately (63).

A pediatrician should be a complete expert in autism, as other professionals are engaged for this purpose, yet dentistry concerns in subjects on the autistic spectrum should improve any effort to face at this issue early in pediatric life.

However, diagnosing autism is particularly burdensome in early childhood due to the complexity of ASD development and etiopathogenesis, which compels physicians to endeavor novel diagnostic strategies (64–71). The topic of oral, dental, and periodontal health in the early pediatric life of ASD subjects is still missing from the correct management of these individuals by pediatricians and pediatric dentistry (72, 73).

What is the role of current pediatric dentists in this context? The level of preparation provided by current schools of specialization in pediatric dentistry, based on the program contents, is adequate to deal with children with ASD, but the number of specialists is still too much small to address the vast problem both at a clinical and scientific level and greater promotion and political-social awareness in this area would be desirable.

Therefore, talking about oral health in ASD subjects involves the ability to diagnose autistic symptoms as early as possible, supporting the pediatric consultation with dentistry professionals such as oral and dental hygienists and dentists, in order to optimally make children's parents aware of their role in the oral hygiene and teeth monitoring of their kids and scheduling periodic visits with experts (72). Once a diagnosis of purported autistic behavior was made, usually the child is in preschool or school, and most efforts are focused on psychological and behavioral issues. Oral hygiene and dental evolution are secondary interests, making the autistic child pay a heavy price for the frequently complex dental care she/he will need.

For example, the genetics of ferroptosis has been suggested as a possible methodology to diagnose autism very early. At least four ferroptosis-related genes (FRGs) have been linked with the likelihood, in the AUC (Area Under the Curve) values of ROC (Receiver Operating Characteristic) curves, of developing autism, particularly if involving the regulatory genes RORA (Related Orphan Receptor A), FAF1 (Fas-associated Factor 1), the microRNA gene miR-4703-3p, NFYB (Nuclear Transcription Factor Y Subunit Beta) and the microRNA miR-6073 (74). Nevertheless, genetics is not so routinely available, whereas neuroimaging might be much more affordable and available (75) and early motor signs (76).

Neonatology should improve its interest in autism pathogenesis and development as well as pediatrics. A recent paper, based on the national Swedish birth registry from 2007 to 2018, assessed that of 383 preterm infants born at a median age of 23.3 weeks, about 24% further developed ASD and 30% attention deficit hyperactivity disorder (ADHD) (77). Many attempts, including the investigation of the immune and cytokine profile of pregnant mothers (78), are in the spotlight. The early diagnosis of the autistic syndrome should encourage a prompt series of consultations and actions to safeguard children's oral health and prevent problems that may compel parents to resort to burdensome dental interventions.

Due to poor or superficial oral hygiene, early tooth decay, often associated with periodontal problems, is frequently due to few or no preventive instructions. This circumstance makes it crucial to envisage possible dental concerns in autistic subjects (79).

Their prevention should be effective by setting up counseling services with dental hygienists in pediatric clinics and supportive structures for families. That would enhance the awareness of pediatricians in the first 1,000 days of life and would bring forward the first dental appointment (with a pediatric dentist and possibly a neuropsychiatrist) along with the dental hygienist and possibly the children's caregivers to soon after an early diagnosis of autism and might even stimulate new multi-media tools to interact even at a distance with the ASD subject.

Telehealth refers to providing prevention, diagnosis, and treatment services through innovative technologies when the health professional and the patient are not in the same place or are, for some reason (e.g., COVID-19 emergency), unable to meet in person.

A critical question, especially in the case of complex pathologies such as ASD, concerns the possibility of carrying out a program remotely as what are the most effective tools both in the diagnosis and intervention phases (80, 81).

Some authors developed a parent-mediated early intervention program for preschool autistic children. They consider autism spectrum disorders as impairments of socio-communicative development and propose a first Denver model, emphasizing the importance of social interaction and the need to promote reciprocity and social imitation (80).

It is increasingly important to understand which are the most proper tools in the diagnostic phase and which can offer good reliability even when the assessment is carried out online; in the same way, it is necessary to identify the most effective early interventions that can also be implemented through a remote rehabilitation process or remote preventive action.

## Prevention of oral and dental disorders in autistic subjects. An update

The role of an early approach in the oral hygiene educational training, consultation, and scheduled visits, cannot only be considered as overly thorough with child's health but significantly preventing severe damage to teeth and reducing the economic burden on public health services and healthcare in general significantly (82). For example, in Europe in 2018, the economic impact of oral diseases, such as periodontitis, caused by poor hygiene monitoring, amounted to 2.52 billion €. In contrast, the indirect cost related to this was considerably higher, i.e., 158.64 billion €, quite comparable to the USA (154.06 billion $) (83). In this context, Italy, with 148.24 billion € in health expenditure and 4.78 billion € for dental expenditure paid directly by patients, stands just after Germany (6.26 billion €), ranking second in the list (83). These data mean that the private expenditure for dental interventions in Italy is very high and requires improvements in the promotion of preventive measures to reduce this burden.

We should add a significant component to this estimation due to incorrect oral hygiene management in children with disabilities, including autism. A possible bias is considering oral health as a general problem for all preschool and school-age children. Autistic subjects do not enjoy much more attention than their neurotypical peers, probably because the autistic subject is tought to understand in terms of behavior and language (84). This would explain why most of the current literature about oral hygiene in autism deals almost exclusively with caries, as with neurotypical children. In contrast, the ASD subject bears a heavier burden if, as outlined above, an early diagnosis has not been made and dental assistance has not previously been offered. So, counseling usually consists of valuable information for all children, for example, reducing sugar intake and frequently brushing teeth.

Dietary sugar exposure is considered the leading cause of caries (as DMFT/dmft ratio) in ASD children, as 85.7% of those subjects consume sugar-enriched snacks in their diet (85). However, a survey from Moorthy et al. on 136 autistic children and 136 neurotypical, healthy children aged 5–12 years in a case-control study reported that there was no significant difference in DMFT/dmft between the two groups, even though ASD children, even with better oral hygiene practice, showed a worse OHI-S score than controls (86). This contradictory evidence may be considered a misleading message. However, it is crucial to state that numerous clinical studies suffer from bias due to the extreme difficulty of managing autistic patients enrolled in the investigation, with concerns regarding their parents, the difficulty in clearly using the correct language and attitude to communicate with the autistic child (84).

In particular, COVID-19 pandemic emphasized the many critical biases in the clinical research on ASD.

Alonso-Esteban et al., reported, in a systematic review, that COVID-19 social confinement is causative of contradictory results in the clinical research, due to ASD severity, age distribution and family hallmarks (87).

The study by Suhaib et al. of 58 Pakistani ASD children matched with 27 siblings without ASD reported that, despite comparable dietary habits and sugars intake, autistic children had a higher incidence of caries (50%) than controls (22.2%), with 24% of dental plaque in ASD children compared to 14% in the control cohort (88).

As mentioned above, some of these studies may be biased due to the interview about children's dietary habits reported by tutors or parents. This fact may occur mainly if the study includes kinship peers as controls. Parents are worried about creating competition between siblings since autistic children receive more attention from adults (89).

The overall impression from many papers dealing with oral and dental hygiene in ASD children is that caries is not always associated explicitly with autistic subjects. However, educational programs for parents, teachers, caregivers, and professionals, emphasize their crucial importance in preventing dental and periodontal damage in children with autism (90). On the other hand, the complexity of the dietary habits of ASD individuals and unusual eating behaviors (for example, keeping a wrapped sugar lollipop in the mouth for several minutes while sucking the toffee underneath the cover through a little hole) is a significant point at issue in the debate about oral hygiene in autism.

So, compared with typically developing peers, autistic subjects have atypical dietary habits, which may dramatically affect the health status of oral, dental, and periodontal structures. Children affected by autism consume higher amounts of simple and refined sugars than neurotypically developing peers, who, moreover, include more raw and cooked vegetables in their diet than ASD subjects (91). This fact, however, is not simply a neurological sign of cognitive or behavioral impairment, leading to typical psychic traits, but might even indicate a more complex role of sugars in the gut-brain microbiome axis (GBMA) in these subjects. Excess to sugars in ASD children may induce hyperactivity (too often, parents do not know this) and promote dental injuries, so a balanced, proper diet for these subjects is crucial (92, 93).

The role of glucose in autism is significant as it affects neuronal migration and neuronal mitochondrial dysfunction, which characterize autism-associated dysconnectivity (94). In addition, a glycan-based language rules the relationship between the brain and gut microbiome (95), a finding that may throw some light on why autistic subjects, having impaired dietary habits, are mainly prompted towards sugars assumptions in their diet (96, 97).

If this is true, it is challenging to prevent caries by sequestering sweet snacks from the ASD subject's diet between meals. Even the proposal to use sweeteners encounters some difficulty, as stevia is expensive and aspartame is noxious. In addition, the high preference for sugar-based snacks, confectionery, and a soft diet in ASD children raises essential concerns about dental prophylaxis, which is necessary to reduce caries and dental injuries in such individuals and to lower the frequency with which parents or tutors have to schedule a dental appointment (98).

A concern in this perspective is crucial to be highlighted, yet.

A further issue is that ASD children not only prefer sweet snacks but show selective diets or “picky” eating habits, exhibiting behaviors that have recently been associated with an aberrant sensory experience, such as heightened reactivity to food tastes and textures (99). Autistic subjects experience a selective or “picky” eating attitude, probably because they suffer from an aberrant sensory perception of tastes, including heightened reactivity to defined tastes and aversion to others, a condition caused by atypical connectivity of the gustatory cortex at a functional level (99).

This finding raises the urgency of recommending that healthcare providers in dentistry to plan and produce goods able to be positively selected and appreciated by ASD children, thus favoring their optimal management and that pediatricians be fully informed about taste impairments in autistic children.

Sugar was preferred more than three times a day between meals (26 out of 35 = 74%), whereas the same proportion refused sugar within a meal (98).

Autistic children should be deceived with appealing foods mimicking sugars to prevent dental caries due to an excess of refined sugars in the diet, but this task is particularly burdensome and tricky because of the complex response of ASD subjects to smelling and tasting (99). Therefore, this issue is of utmost importance in preventing dental injuries in autism.

Carli et al. reported a prevention program involving 100 autistic patients (78 males and 22 females) to improve dental health by evaluating some parameters such as plaque index, gingival index, the caries index DMFT/dmft, the frequency with which subjects brushed their teeth and the frequency of snack intake (100). Even the kind of toothpaste, i.e., their smells and tastes, should be included in such parameters to be investigated.

Some years ago, Jaber found in 61 ASD patients matched with 61 neurotypical children that autistic individuals suffered from a higher prevalence of caries, poor oral hygiene, and frequency of unmet needs for dental counseling and intervention (101).

The general message we could extract from this evidence is that rather than preventing children from eating sweet snacks, increasing the number of dentist examinations, it seems less traumatic if the examinations are conducted properly. In addition, a closer interaction between parents and dentists is crucial for dental prevention in autism (102).

Furthermore, autistic subjects have impairments in sensory perception. The sensory processing approach for treating autistic subjects in dentistry dates back to Kanner's work in 1943, describing subjects who fear noises from devices and machines, flickering lights, and mouthing objects (103, 104).

Some years later, Bergman and Escalona reported the first hypothesis about sensory-based impairment in autism, suggesting that the unusual behavior of autistic children towards novel lights, sounds, and smells reflected a defensive attitude (105). ASD children have been found to exhibit a complex pattern of aversive and scared behaviors against new sensory inputs, showing difficulty in sensory processing (106–111).

This evidence suggests that professionals should thoroughly understand sensory processing in autism to improve dental examinations (112, 113). A study by Stein et al. reported that in the ASD group compared to the “other disabilities” group, about 46.4% vs. 32.1% showed difficulty in getting their teeth brushed or cleaned by the dentist. Also, 48.3% vs. 28.9% exhibited difficulties in opening their mouths, cooperating, and being quiet without screaming, 63.0% vs. 49.2% have parents who believe that the sensory sensitivity of their children interfered with dental processes and that 44% vs. 51% did not plan any dental appointment within one year (112). A possible consented weekly program until proper oral hygiene is acquired, may include scheduled examinations every 15, 30, or 60 days, depending on the child's problem, telehealth availability, and the presence of a home care assistant.

Searching for appropriate and optimal cooperation between families and dentists by adopting new strategies to improve the management and frequency of dental examinations of autistic subjects may represent the simplest solution to overcome the enormous difficulty of treating autistic children.

Furthermore, it may prove possible to use virtual technology and multi-media devices to manage the best possible behavioral relationship with the autistic subject to achieve correct oral hygiene (53, 114–116).

## Besides oral caries. Malocclusion, bruxism, periodontal and gingival concerns in ASD

Although oral caries and oral hygiene represent the primary concern in ASD children, due to their enormous difficulty in being treated by a dentist, even in different European countries (53), periodontal and gingival diseases in autistic people are widespread (20).

However, recent evidence failed to show a fundamental difference in some defects such as malocclusion and bruxism in ASD subjects compared with non-ASD ones. Some recent meta-analyses reported that the risk of having malocclusion was comparable in ASD subjects and their neurotypical counterparts (16), yet in autistic subjects, this orthodontic concern is problematic due to scant compliance (27) and maybe connective tissue peculiarities (26).

In Italy, for example, a study by Bagattoni et al. of 64 ASD children matched with 64 neurotypical peers reported that autistic subjects showed significant (*p* < 0.01) dental trauma, bruxism and habits of biting objects, higher rates of dental plaque, higher DMFT/dmft and anterior open bite. In contrast, fluoride exposure and enamel defects were lower or comparable to controls (117).

The same uncertainty was reported for bruxism, despite ASD subjects showing a higher likelihood of developing bruxism than controls (18). A paucity of available data probably causes this bias, as managing autistic subjects, even for research purposes, is particularly difficult.

In any case, some steps forward have been accomplished.

Several years ago, Johnson et al. suggested some approaches to prevent gingival injuries and illnesses in autistic subjects (118). Many of these injuries are described as factitial, caused by self-injurious behavior (SIB) (119). SIB is still found even among neurotypical children with psychological or relationship problems, not simply in autistic subjects; therefore, SIB is a widespread hallmark of children with social and adaptability concerns, not a matter exclusively associated with autism (120–122).

Dealing with ASD subjects needs expert caregivers and health personnel to be endowed with sound methodologies and visual pedagogy (17, 123).

A recent meta-analysis of oral health in autistic subjects reported that most papers deal with the DMFT/dmft index, and only one-third with plaque, gingival status, and salivary pH (12). Noticeably, ASD children showed higher DMFT index, plaque index and gingival index, and lower pH than neurotypical peers, so assessing the evidence that autistic children have poor oral health and a higher risk of developing caries and teeth injuries (12), despite some controversial opinion (124).

The management of an autistic subject requires a highly expert series of methodological criteria in order to facilitate the relationship with dentistry professionals, such as dentists and oral hygienists, psychologists or neuropsychiatrists, and pediatricians, all supporting and expert figures particularly important in helping ASD children's parents or tutors support teachers (caregivers) to oral hygiene practice (100, 114, 125–129), even considering differential habits and behaviors between males and females (130).

Different nutritional habits are another confounding factor in clinical reports about ASD children. Any controversial result may be explained by considering the complex participation in the oral health of an autistic subject of dietary and GBMA (gut-brain microbiome axis) impairment, imbalanced immunity, the apprehensive attitude of parents towards their children's dental care, and much else, including impairments in the oral and tongue microbiomes (131–134).

Therefore, when discussing the oral microbial and immune micro-environment in an autistic subject, many contradictory factors must be considered before associating any oral health impairment with incorrect hygiene. Both incorrect hygiene and an unbalanced diet contribute to the pathological onset of dental injuries.

Incidentally, dental injuries often require complicated dental interventions, particularly in ASD subjects, who must undergo complete sedation or general anesthesia (135).

Furthermore, a significant periodontal ailment, gingivitis, may be associated with particular genetic determinants and single-nucleotide polymorphism (SNP) (136). Different genes and SNP are associated with the phenotypic traits of the autistic subject (137, 138), and salivary markers in ASD are widely used to investigate the genetic landmarks of ASD (46).

Therefore, periodontal diseases in autistic subjects have a broad spectrum of etiological causes besides oral hygiene and should be diagnosed with particular caution before associating them with inadequate daily brushing and cleaning of teeth.

Diet, genetics, and oral health habits cause periodontal disease and tooth injuries in ASD children (139). In addition, plaque and bacterial biofilms in an altered oral microbiome are formidably linked with altered oral immunity due to impaired composition and functionality of the gut microbiome (140).

The complexity of autism dentistry is not solely of a behavioral nature but includes many factors, including dietary habits, nutritional intake, oral and gut microbiomes, and genetics.

Considering all these issues and concerns, which message can we forward to dentists and ASD children's parents?

First, public health policy should stress prevention and therapy programs for children with disabilities, particularly autism, starting early in the first 1,000 days of life.

As is well known, some reports have assessed that ASD children have more dental health problems than others.

Given this situation, public healthcare strategies should be significantly promoted, starting with a Nationwide Survey of the available healthcare services for ASD in Italy (141).

Second, ASD children are usually considered as a whole, whereas boys and girls show different impacts on oral health and should be considered separately (142). Autistic boys and girls show differences in plaque index, caries prevalence, different mean dmft and DMFT, different drooling of saliva, deep palate, a habit of thrusting their tongue, tooth wear, bruxism, delayed eruption, and so on (18, 117, 127).

Third, dental indoor environments and dentistry devices should probably be adapted to be “autism-friendly.” Politicians should promote legislation and educational programs like those for people with motor or other disabilities. New strategies have been recently suggested (127).

Fourth, autism should be considered a pathology with a substantial social impact to relieve the almost exclusive burden on the family and promote political proposals to sensitize public opinion. In this case, dentistry for ASD children would be not simply the commendable enterprise of a few appreciated professionals but a good hallmark of our modern civilization.

Autistic children need a considerable amount of health attention for their oral health management.

## Conclusions and future remarks

The overall landscape of ASD dentistry is particularly complex. Our overview and bias analysis have reported that much has to be done, yet, particularly in sensitizing professionals engaged in dentistry of these children. The AMSTAR-2 check list (Table 2) reports that our study did not perform a meta-analysis of our search, due to the high presence of heterogeneity.

**Table 2:** AMSTAR 2: a critical appraisal tool for systematic reviews that include randomised or non- randomised studies of healthcare interventions, or both.


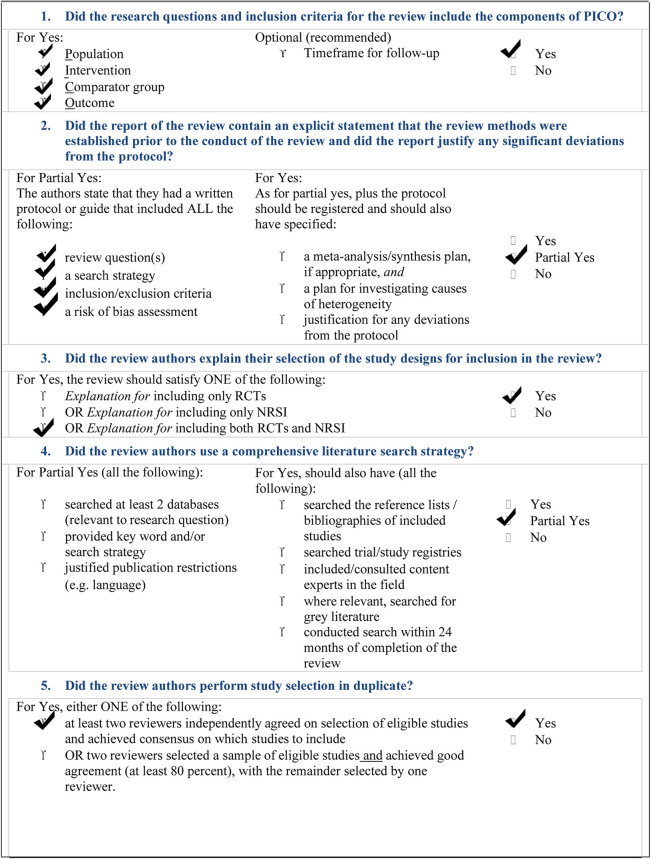
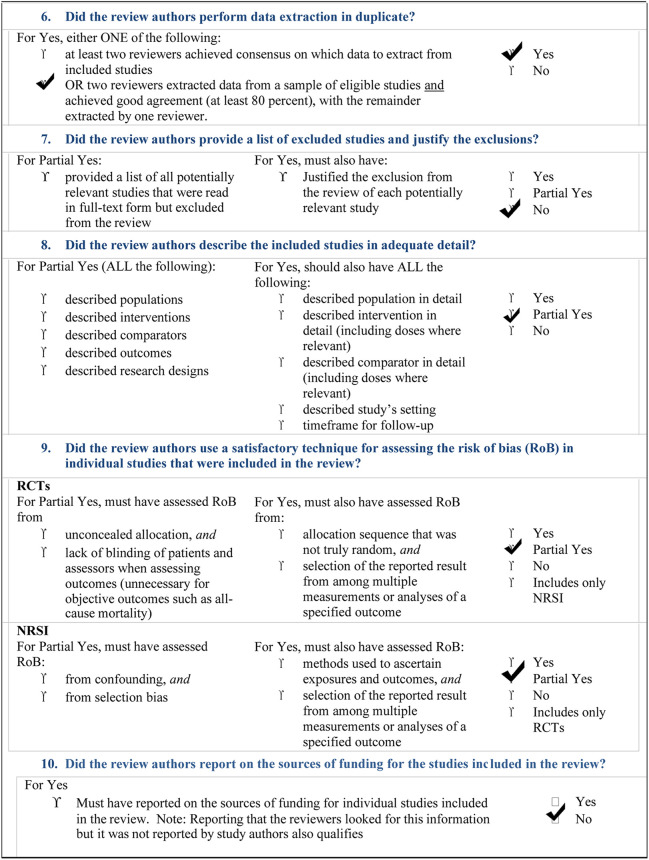
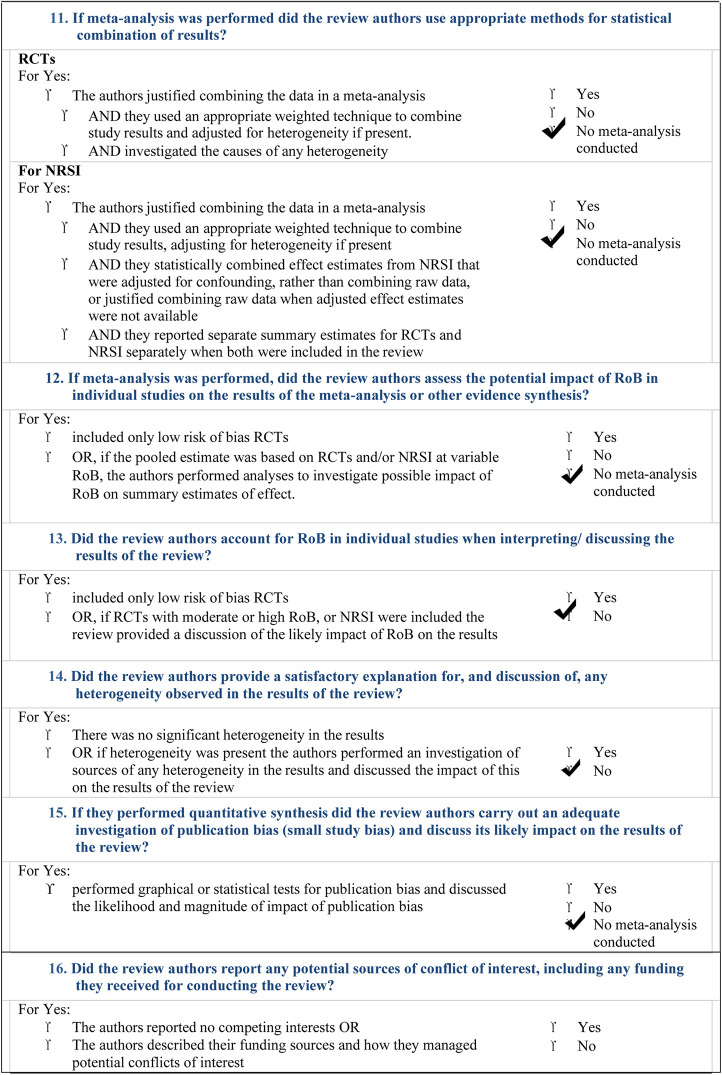

We tried to describe the different approaches of autistic children in dentistry, and this should sensitize physicians, dentists, health professionals, politicians, parents, and tutors to promote sound suggestions to overcome the difficulties of oral health in these complex subjects. Oral and dental sciences should improve research and technological innovations. The dentist should treat the autistic child with the respectful and competent attitude she/he uses for non-autistic children. Nevertheless, she/he should consider autism a complex syndrome, where the professional ability of the dentist compels her/him to adopt new strategies to treat the problematic child, often alongside with children's parents.

We want to highlight some crucial standpoints and conclusive landmarks on this subject from the literature, mediated by our clinical experience, to date:

(a) A very early diagnosis of autism should help professionals to better approach the significant problems of oral health in the autistic child by planning a proper preventive schedule with recommendations. Even neonatology should improve its interest in ASD dental health;
(b) Parents and tutors should not be left alone in managing their children's oral health. Neuropsychiatrists and pediatricians, along with other caregivers, should actively join the activity of pediatric dentists by training and assisting dental activity *via* education, information, and promotional proposal and actions, as previously described in the text;
(c) Continuous updating on ASD is crucial to be properly and thoroughly informed about how better to manage the autistic child in the dental clinic and services;
(d) The active participation of healthcare companies of dental biomedicals in improving products and devices for autistic children and very young patients with disabilities are crucial, and politics should encourage this;
(e) Parents must be continuously trained, educated, and assisted in managing their children's oral health by warnings about diet, information about available toolkits, dentists providing telemedicine, visual technology, and specific professionals.
(f) Individual periodic dental appointments and followed up by dentists should be promoted as personalized and not scheduled as with neurotypical peers;
(g) Preventive meetings and talks, or even interviews, with neuro-psychiatrists, joining pediatricians and dentists, as well as parents, is crucial to define a series of recommendations and actions prior to any dental interventions on ASD children, including oral health programming.
(h) The use of music as a therapeutic support tool must be strongly encouraged

The autistic subject is a frail individual needing particular care and attention by institutional health services, professionals, caregivers, and parents in managing oral health, preventing economic burdens and social concerns, and avoiding unnecessary further suffering for complex dental therapies involving hospitalization, as unfortunately still happens.

For this reason, research has to be improved and professionals continuously trained.
